# Mapping the Potential Risk of Mycetoma Infection in Sudan and South Sudan Using Ecological Niche Modeling

**DOI:** 10.1371/journal.pntd.0003250

**Published:** 2014-10-16

**Authors:** Abdallah M. Samy, Wendy W. J. van de Sande, Ahmed Hassan Fahal, A. Townsend Peterson

**Affiliations:** 1 Biodiversity Institute and Department of Ecology and Evolutionary Biology, University of Kansas, Lawrence, Kansas, United States of America; 2 Entomology Department, Faculty of Science, Ain Shams University, Abbassia, Cairo, Egypt; 3 Erasmus Medical Center, Department of Medical Microbiology and Infectious diseases, Rotterdam, The Netherlands; 4 Mycetoma Research Centre, University of Khartoum, Khartoum, Sudan; University of Tennessee, United States of America

## Abstract

In 2013, the World Health Organization (WHO) recognized mycetoma as one of the neglected tropical conditions due to the efforts of the mycetoma consortium. This same consortium formulated knowledge gaps that require further research. One of these gaps was that very few data are available on the epidemiology and transmission cycle of the causative agents. Previous work suggested a soil-borne or *Acacia* thorn-prick-mediated origin of mycetoma infections, but no studies have investigated effects of soil type and *Acacia* geographic distribution on mycetoma case distributions. Here, we map risk of mycetoma infection across Sudan and South Sudan using ecological niche modeling (ENM). For this study, records of mycetoma cases were obtained from the scientific literature and GIDEON; *Acacia* records were obtained from the Global Biodiversity Information Facility. We developed ENMs based on digital GIS data layers summarizing soil characteristics, land-surface temperature, and greenness indices to provide a rich picture of environmental variation across Sudan and South Sudan. ENMs were calibrated in known endemic districts and transferred countrywide; model results suggested that risk is greatest in an east-west belt across central Sudan. Visualizing ENMs in environmental dimensions, mycetoma occurs under diverse environmental conditions. We compared niches of mycetoma and *Acacia* trees, and could not reject the null hypothesis of niche similarity. This study revealed contributions of different environmental factors to mycetoma infection risk, identified suitable environments and regions for transmission, signaled a potential mycetoma-*Acacia* association, and provided steps towards a robust risk map for the disease.

## Author Summary

WHO has recognized mycetoma as one of the neglected tropical diseases (NTDs) worldwide. Studies indicate infections from soil or possibly mediated by thorn pricks, but no detailed studies have investigated effects of soil type and *Acacia* distributions on mycetoma in Sudan. Here, we investigated risk factors associated with mycetoma infections in Sudan using ecological niche modeling (ENM), integrating mycetoma case records, *Acacia* records, and geospatial data summarizing soil, land-surface temperature, and greenness. ENMs calibrated in endemic districts were transferred across Sudan, and suggested that greatest risk was in a belt across central Sudan. Mycetoma infections occur under diverse environmental conditions; we found significant niche similarity between *Acacia* and mycetoma. Model predictions were amply corroborated by a preliminary assessment of a much larger mycetoma case-occurrence data base. Our results revealed contributions of different environmental factors to mycetoma risk, raised hypotheses of a causal mycetoma-*Acacia* association, and provide steps towards a robust predictive risk map for the disease in Sudan.

## Introduction

Mycetoma is a chronic, devastating, inflammatory disease of the subcutaneous tissues that spread to involve the skin, deep structures and bones, and is characterized by deformity, destruction and disability especially in late stages [1]–[3]. Etiological agents are identified by culturing their characteristic compact mycelial grains [4], [5]. The infection most often affects the lower extremities of individuals living in developing tropical and subtropical countries [6]. Two forms of mycetoma have been identified [3], [7]: actinomycetoma caused by a group of filamentous bacteria, and eumycetoma caused by any of 30–50 species of hyaline and pigmented fungi [4], [8]–[11].

The organisms causing mycetoma are geographically distributed worldwide, but are particularly common in tropical and subtropical areas, in the so-called ‘mycetoma belt,’ which includes Mexico, Venezuela, Mauritania, Senegal, Chad, Ethiopia, Sudan, Somalia, Yemen, and India [11]. The incidence and geographic distribution of mycetoma are underestimated, as the disease is usually painless and slowly progressive, such that it is presented to health centers only in late disease stages by most of patients; it is not a reportable disease [12]–[14]. Mycetoma is a socioeconomically biased disease, and typically appears in low-income communities with poor hygiene; for example, agricultural laborers and herdsmen appear worst affected [15], [16]. Studies revealed that minor traumas can allow pathogens to enter the skin from the soil [7], or through *Acacia* thorns, to the point that *Acacia* thorns have been found embedded in mycetoma lesions during surgery [4], [17]. Fungal infections responsible for eumycetoma in Sudan are predominantly caused by *Madurella mycetomatis* [4].

Studies to date suggest a soil-borne or thorn-prick-mediated origin of mycetoma infections [4], having demonstrated *M. mycetomatis* DNA on *Acacia* thorns and in soil samples [4]. Although prevailing thought is that the soil is the ultimate reservoir for mycetoma infections, attempts to culture the fungus from soil samples have failed [4], [14]. A more recent study suggested that cattle dung may play a significant role in the ecology of *Madurella*, based on the observation that *M. mycetomatis* is phylogentically closely related to dung-inhabiting fungi [18].

Mycetoma ranks among the most neglected diseases worldwide, to the point that it was omitted even by major neglected tropical disease (NTD) initiatives across the globe [19]–[21]. Recently, mycetoma was added to the WHO's list of NTD priorities [11]. The known geographic distribution of mycetoma etiological agents shows intriguing variation with respect to environmental factors [22]: they occur in arid areas with a short rainy season, and extreme conditions have been suggested as a prerequisite for survival of the causative organisms [22]. Still, the geographic distribution of the disease remains in large part uncharacterized. In this paper, we report explorations using ecological niche modeling to (1) estimate the current niche and potential distribution of mycetoma in an important endemic region (Sudan), (2) investigate risk factors associated with mycetoma infections in Sudan and South Sudan as reflected in distributional associations with environmental features, and (3) test *Acacia*-mycetoma associations based on overlap of the ecological niche of mycetoma infections with that of trees of the genus *Acacia*.

## Materials and Methods

Occurrence records for mycetoma cases were obtained from published scientific literature via the PubMed database (www.ncbi.nlm.nih.gov/); we also used mycetoma data deposited in the GIDEON database (http://www.gideononline.com/). Studies were selected if they described positive mycetoma cases, and were referred to specific geographic locations that could be georeferenced precisely. When geographic references were textual in nature, latitude-longitude coordinates were assigned via reference to electronic gazetteers (e.g., http://www.fallingrain.com; [23]), and Google Earth (www.earth.google.com/); 11 records were obtained by georectification and georeferencing of Figure 1 from Ahmed *et al.* 2002 [4], [17], [24]. We eliminated duplicate records and records presenting obvious errors of identification prior to any further analysis.

**Figure 1:**
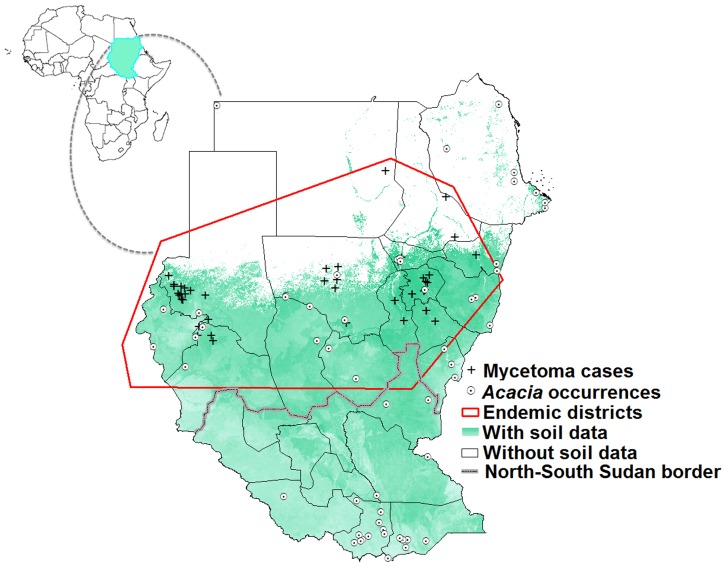
Geographic distribution of mycetoma cases and *Acacia* trees across Sudan and South Sudan (crosses and dotted circles, respectively). Some areas across the region (in white) were not included in some analyses for lack of data on soil characteristics.

Occurrence records were obtained for *Acacia* from the Global Biodiversity Information Facility (www.gbif.org) to test contributions of the trees to a robust mycetoma model for Sudan and South Sudan [4], [17], [24]. We filtered *Acacia* occurrences to include only Sudan and South Sudan. All duplicate records and records lacking georeferences were excluded from analysis.

To characterize environmental variation across Sudan and South Sudan, 8-day composite Land Surface Temperature and monthly Normalized Difference Vegetation Index (NDVI) data were drawn from Moderate Resolution Imaging Spectroradiometer (MODIS) satellite imagery at 1 km spatial resolution. We also used 10 variables from the World Soil Information site (http://www.isric.org) to summarize chemical and physical soil characteristics (Supplementary file S-1). Soil data were obtained for each of 2 depths for each variable: 0–5 cm and 5–15 cm. Soil variables represented a collection of updatable soil property and class maps of the world at 1 km resolution produced using model-based statistical methods, including 3D regressions with splines for continuous properties and multinomial logistic regression for classes [25].

LST and NDVI data were downloaded for 2005–2011 from the Land Processes Distributed Active Archive Center data holdings, using the NASA Reverb Echo data portal (https://reverb.echo.nasa.gov/reverb/) as described in greater detail elsewhere [26]. The LST product has been validated via several ground-truth and validation efforts over widely distributed locations and time periods [27]. The NDVI product has been used broadly for monitoring vegetation conditions and land cover change [28]. We calculated grids for the minimum, maximum, median, and ranges of values for LST and NDVI across the entire time sequence for all of Sudan and South Sudan to provide a rich characterization of environments across the country.

The Grinnellian fundamental ecological niche is defined by the set of coarse-grained, non-interactive environmental conditions under which a species is able to maintain populations without immigrational subsidy [29]. ENM attempts to estimate these niches from incomplete information by relating known occurrence locations and the environmental values that they present to the broader environmental landscape. This approach was used to relate known mycetoma occurrences to raster environmental data in an evolutionary-computing environment; in this case, a maximum entropy algorithm (MaxEnt v.3.3 [30]) was used to estimate ecological niches both for *Acacia* spp. collectively and for mycetoma. Niche model outputs for *Acacia* were in turn used as input in calibrating models for mycetoma; in the end, we developed models based on LST/NDVI and all combinations of soil and *Acacia* information, and the *Acacia* models were calibrated with and without soil information. Accessible areas (**M**) for mycetoma and *Acacia* were assumed to include all of Sudan and South Sudan, based on their wide geographic distributions. We calibrated ENMs across a subset of the study region corresponding to known endemic districts; models were then transferred across all of Sudan and South Sudan for interpretation; for comparison, we also calibrated models across all of Sudan and South Sudan (i.e., not just known endemic districts), although the model transfer approach should be more rigorous [31]. ENMs outputs were converted to binary maps using a least training presence thresholding approach adjusted to admit 5% (*E* = 5%) error rates [32].

To test the ability of the ENM algorithm to predict occurrences accurately across unsampled areas of Sudan and South Sudan, we used a partial receiver operating characteristic (ROC) approach [32]. This approach evaluates models only over a range of relevant predictions, and potentially allows differential weighting of omission and commission errors, and therefore is preferable to traditional ROC approaches [32]. Models were evaluated by calibrating models with a random 50% of occurrences, and comparing the threshold-independent area under the curve (AUC) to null expectations. To compare partial ROC AUC ratios of each model with null expectations, the dataset was bootstrapped, and probabilities obtained by direct count, with AUC ratios calculated using a Visual Basic script developed by N. Barve (University of Kansas), based on 100 iterations and an *E* = 5% omission threshold.

As a further, and more rigorous, test of model predictivity, we derived a preliminary view of mycetoma case data archived in the Mycetoma Research Center, in Sudan, based on cases from 1991–2014. In view of the large scale of this data resource, we selected and georeferenced ∼10% of the overall data archive at random; we eliminated cases lacking geographic references and removed records from duplicate localities, which left 158 localities for this preliminary analysis. We assessed the relationship of these data to the best of our model predictions via a one-tailed cumulative binomial probability calculation that assessed the probability of obtaining the observed level of correct prediction by chance alone, given the background expectation of correct prediction based on the proportional coverage of the region by the prediction [29].

Background similarity tests [33] were used to assess similarity between models of niches of *Acacia* and mycetoma. We first reassessed the accessible area (**M**) for both species [34]: mycetoma is limited approximately to the belt between the latitudes of 15°S and 30°N [7], [20], and *Acacia* is widely distributed and grows in a wide range of habitats [35], so we can set **M** as all of Sudan and South Sudan, or alternatively as only the known mycetoma-endemic districts (Figure 1). To test the null hypothesis of niche similarity between mycetoma and *Acacia* against the backgrounds of their respective **M** hypotheses [34] as described above, we used *D*-statistics and Hellinger's *I* implemented in ENMTools [33]. We tested niche similarity with respect to two environmental data sets: (1) LST and NDVI; and (2) LST, NDVI, and soil characteristics. The background similarity test is based on models of random points from across the accessible area in numbers equal to numbers of real occurrence data available for each species in the study, with 100 replicate samples. The null hypothesis of niche similarity was rejected if the observed *D* or *I* values fell below the 5^th^ percentile in the random-replicate distribution for comparison of the ENMs for the pair of species in question [33].

## Results

We assembled a total of 44 records of mycetoma cases from sites across Sudan (Figure 1). Cases were from North Darfur (14), Gezira (8), North Kordufan (6), South Darfur (4), Sennar (3), and White Nile (3), Khartoum (2), River Nile (2), Kassala (1), and Northern (1) states. Sampling for mycetoma was focused in these regions, which can be considered as endemic districts for mycetoma. Mycetoma cases concentrated in a belt between 12°S and 19°N latitude, with only a few cases outside this area in Sudan. Records for *Acacia* trees were obtained from 59 localities across Sudan and South Sudan (Figure 1). *Acacia* records were not limited to any particular sub-region, but rather were distributed across much of the country. The geographic distributions of *Acacia* trees and mycetoma cases appeared to overlap only in central Sudan. However, *Acacia* is also present in South Sudan, where no records were available for mycetoma.

**Figure 2:**
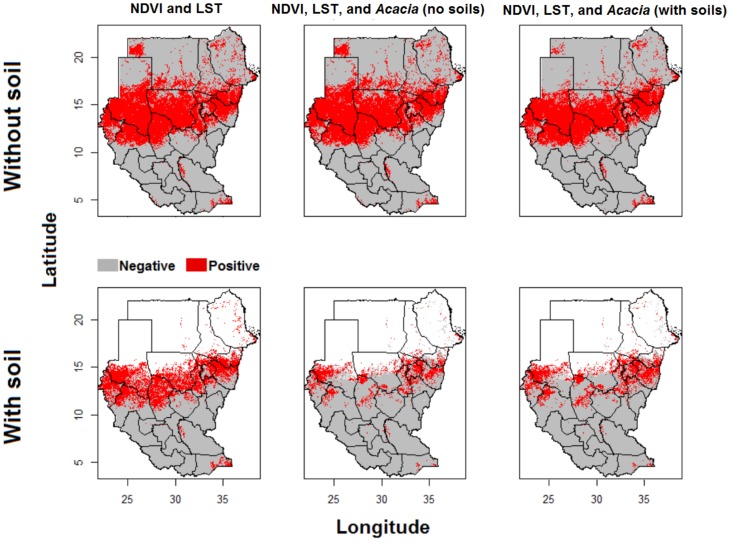
Potential mycetoma distribution based on occurrences in endemic districts. Potential distributions of mycetoma were based on different environmental variables; models were calibrated in mycetoma-endemic districts, and transferred across all of Sudan and South Sudan. White areas have no soils data, and therefore have no model predictions.

**Figure 3:**
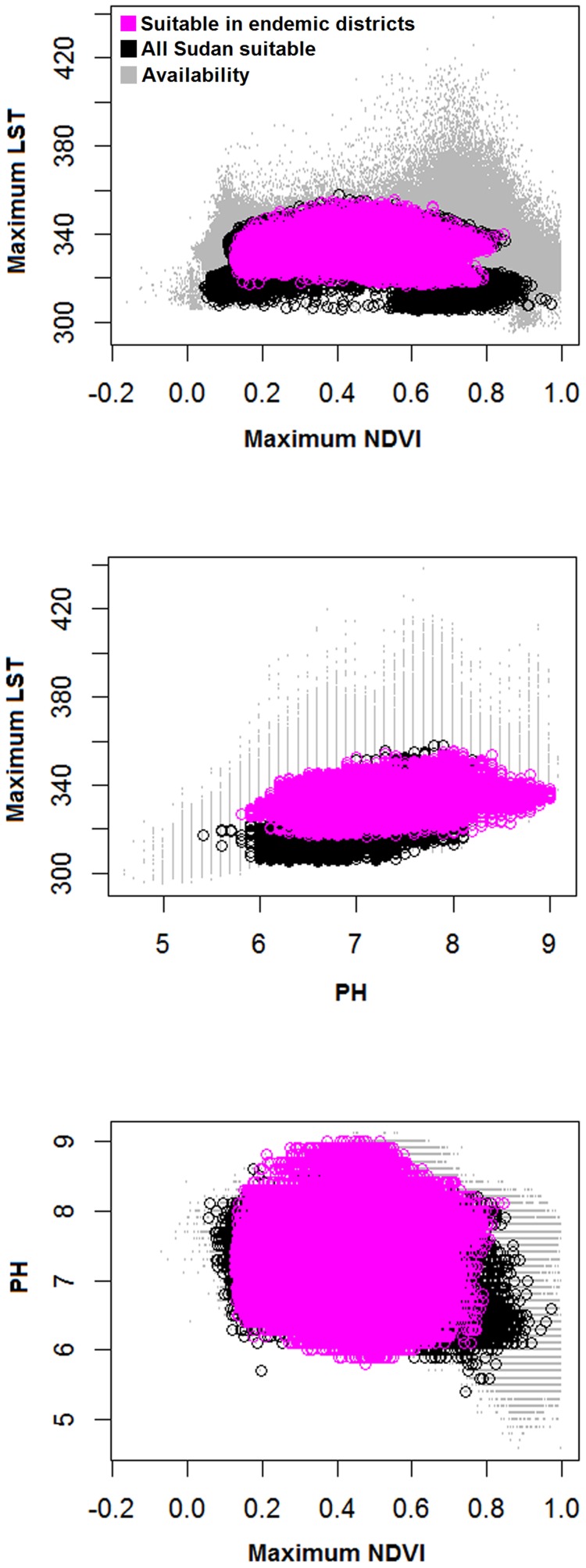
Visualization of mycetoma ecological niches (i.e., the set of environmental values under which the species can potentially maintain populations) in two-dimensional environmental spaces based on different environmental variables. The diagram shows the entire environmental availability across Sudan and South Sudan (light gray color), and conditions identified as suitable across Sudan and South Sudan (black color) and across endemic districts (pink).

We developed models of mycetoma cases based on (1) ENMs calibrated in endemic districts, then transferred to all of Sudan and South Sudan (Figure 2), and (2) ENMs calibrated directly across all of Sudan and South Sudan; these latter models are not depicted in this publication, but are presented in the supplementary materials (S-2). ENMs for mycetoma based on different environmental scenarios were all statistically robust (all AUC ratios uniformly above 1.0 so all *P*<0.01; see Table 1). The model based on all environmental data (LST, NDVI, soils, and *Acacia* distribution) had the highest partial AUC ratios, and thus appeared to perform best. Mycetoma ENM predictions indicated a band of highest environmental suitability in central Sudan between 11°S and 17°N latitude (Figure 2). However, distinct areas were predicted as suitable for mycetoma occurrence elsewhere in Sudan and South Sudan: ENMs based on LST, NDVI, and soil identified a more southerly version of the “mycetoma belt.” High-risk states identified by the ENMs included Kassala, Gedarif, Gezira, Khartoum, Sennar, White Nile, North Kordufan, West Kordufan, South Darfur, North Darfur, and West Darfur. To visualize ecological niches for mycetoma, we linked ENM predictions to characteristic of the environmental landscape (Figure 3): mycetoma occurs on diverse landscapes under wide ranges of environmental conditions, which is to say that no clear and distinctive environmental correlates could be discerned.

**Table 1:** Partial AUC ratios of mycetoma ecological niche models based on different environmental data sets, showing median.

Environmental variables	AUC ratio
LST+NDVI	1.2923 (1.2917–1.8373)
LST+NDVI+soil	1.6864 (1.5179–1.9600)
LST+NDVI+*Acacia* (based on LST and NDVI)	1.3878 (1.3203–1.9011)
LST+NDVI+*Acacia* (based on LST, NDVI, and soil)	1.6518 (1.5767–1.8618)
LST+NDVI+soil+*Acacia* (based on LST and NDVI)	1.8402 (1.7683–1.9924)
LST+NDVI+soil+*Acacia* (based on LST, NDVI, and soil)	1.6365 (1.6012–1.8077)

**Figure 4:**
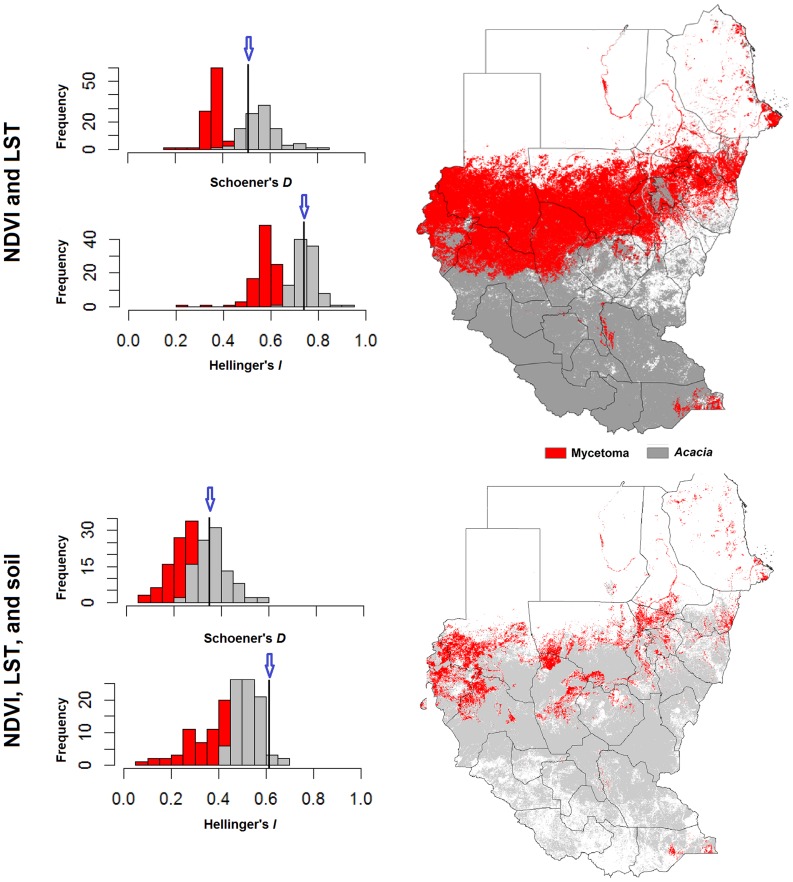
Background similarity test of similarity between mycetoma and *Acacia* ecological niches across Sudan and South Sudan. Niche overlap values were based on Hellinger's *I*, and Schoener's *D* metrics of similarity. Observed values are shown as black line with a blue arrow; null distribution is shown as a histogram.

Neither of the tests comparing niches of mycetoma and *Acacia* was able to reject the null hypothesis of niche similarity (*P*>0.05 in both cases; Figure 4) which is to say that models for mycetoma and *Acacia* were not more different from one another than either was from models based on the background (i.e., across **M**) of the other species. *Acacia* is distributed broadly across Sudan and South Sudan, whereas mycetoma infections were found only in central Sudan, but these results suggest that range difference does not reflect niche differentiation between the two (sampling, diagnostic, and reporting biases may affect the mycetoma data).

**Figure 5:**
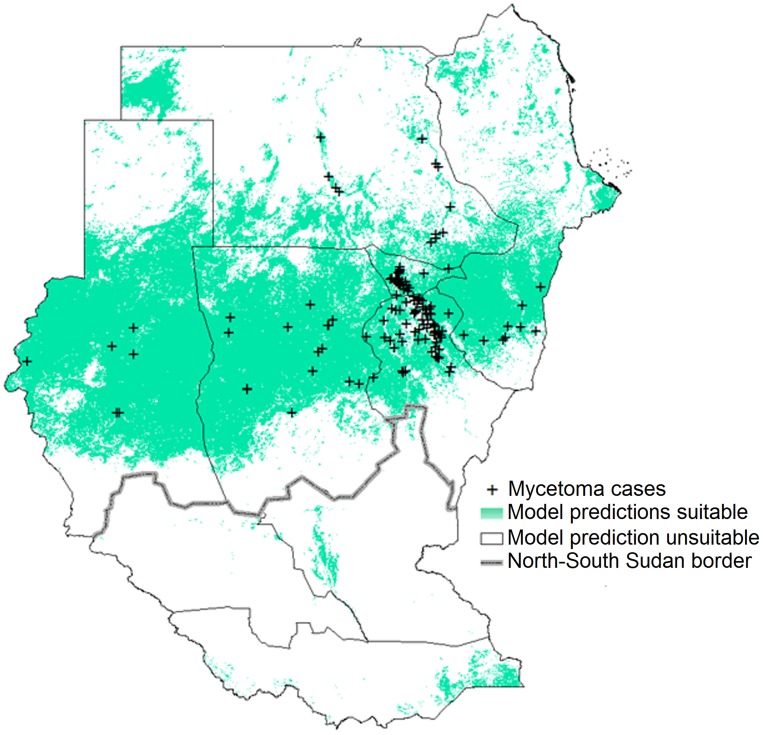
Coincidence between ecological niche model predictions based on LST, NDVI, soils, and *Acacia* (the latter based on LST and NDVI only) with the independent additional case data from the Mycetoma Research Center.

The coincidence between model predictions and the independent additional case data from the Mycetoma Research Center was impressive (Figure 5), such that 149 of 158 of those additional occurrence points were successfully predicted by the model. Model success in anticipating these independent data was statistically significantly much better than random expectations (one-tailed cumulative binomial test; *P*<<0.05).

## Discussion

Known since the 1600s [36] and described more formally in 1842, mycetoma was initially called Madura foot [37]. Mycetoma was subsequently reported in countries presenting diverse environments: Mexico, Venezuela, Mauritania, Senegal, Chad, Ethiopia, Sudan, Somalia, Yemen, and India [11], [14]. Although thousands of cases have been recognized annually, risk factors remain poorly characterized [14], and the mode of transmission remains unknown [14]. Research on mycetoma leaves several hypotheses untested; improved understanding in each respect could reduce numbers of case, improve case outcomes, and offer possibilities for better disease control. Here, we used a new approach, termed ecological niche modeling, which relates case occurrences to environmental characteristics across a relevant region to create a model of the environmental ‘envelope’ (analogous to a coarse-grained definition of the ecological niche) for the species; this niche model allows, in turn, identification of potentially suitable areas for the species to be distributed. Ecological niche modeling has been used previously to understand geographic dimensions of a number of neglected tropical diseases [26], [38], [39], including fungal pathogens [40], [41].

We used ENM to identify suitable sites for mycetoma infections based on environmental predictors, including dimensions thought to be associated with mycetoma cases in previous studies in Sudan [4], [20]. All ENMs indicated high suitability across central Sudan, which appears consistent with cases reported subsequently [17], [42], [43]. It is worth noting that numerous cases reported by the Mycetoma Research Center (MRC) [4] came from the same belt identified by ENMs developed here, and yet had no involvement in our model calibration, providing important corroboration of the model predictions.

Several recent studies have attempted to understand modes of entry and transmission of mycetoma [4], [44], [45], but how people become infected with the causative agents remains unclear [14]. These studies have proposed that the primary reservoir of the causative agents is soil or *Acacia* thorns [4], and that transmission occurs by contact with the causative agent [4], [15], based on observations that mycetoma infections occurred under poor conditions, in agriculturalists and villagers in endemic districts [46], [47]. Our ENMs used soil data, but the causative agent has been identified from areas signaled unsuitable in the soil-based ENMs [4]. Incorporating *Acacia* distributions in models improved predictions, indicating possible relevance of an *Acacia*-mycetoma association.

*Acacia* may thus prove to play some role as a determinant of mycetoma distributional patterns across Sudan and South Sudan, although our results are correlational only and do not provide a direct test of this association. Our background similarity tests between ENMs for *Acacia* and mycetoma could not reject the hypothesis of similarity of the niches of the two species, thus at least not providing evidence against an association, and our models had greatest predictive power regarding mycetoma cases when *Acacia* distributions were included as environmental predictors. The important question remaining, however, is how the causative agent contacts humans, penetrates the skin, and initiates infections.

Previous studies confirmed presence of *Madurella mycetomatis* DNA in 17 of 74 soil samples and in one of 22 thorn samples [4]. Interestingly, attempts at culturing the fungi from these samples failed [4]. Hence, that the study found DNA of *M. mycetomatis* in both soil and thorn samples is of unclear importance, although perhaps culture methods are relatively insensitive or ineffective. In sum, then, our results revealed contributions of different environmental factors to mycetoma risk, identified areas suitable for mycetoma emergence, farther raised the possibility of a mycetoma-*Acacia* association, and provided steps towards a robust predictive risk map for the disease.

## Supporting Information

Text S1 **The variables of the soil characteristics used in model calibration for mycetoma and** ***Acacia*** **spp. in Sudan.** Data downloaded from the World Soil Information (http://www.isric.org). Each variable is available in 2 depths (0–5 cm and 5–15 cm).
(DOC)

Text S2 **Potential mycetoma distribution based on occurrences across all of Sudan.** These models were calibrated across all of Sudan directly based on all records collected from scientific literature and environmental variables for all of Sudan.
(DOC)

## References

[1] Davis JD, Stone PA, McGarry JJ (1999) Recurrent mycetoma of the foot. J Foot Ankle Surg 38 1: 55–60. 10028471 10.1016/s1067-2516(99)80089-1

[2] Pilsczek FH, Augenbraun M (2007) Mycetoma fungal infection: multiple organisms as colonizers or pathogens? Rev Soc Bras Med Trop 40 4: 463–465. 17876471 10.1590/s0037-86822007000400017

[3] Alam K, Maheshwari V, Bhargava S, Jain A, Fatima U, et al (2009) Histological diagnosis of madura foot (mycetoma): a must for definitive treatment. J Glob Infect Dis 1 1: 64–67. 20300390 10.4103/0974-777X.52985 PMC2840937

[4] Ahmed A, Adelmann D, Fahal A, Verbrugh H, van Belkum A, de Hoog S (2002) Environmental occurrence of *Madurella mycetomatis*, the major agent of human eumycetoma in Sudan. J Clin Microbiol 40 3: 1031–1036. 11880433 10.1128/JCM.40.3.1031-1036.2002 PMC120253

[5] Faqir F, Rahman Au (2004) Mycetoma: a local experience. J Postgrad Med Inst 18 2: 172–175.

[6] Sahariah S, Sharma AK, Mittal VK, Yadav RV (1978) Mycetoma of lower extremity. J Postgrad Med 24 2: 113–116. 722604

[7] Lichon V, Khachemoune A (2006) Mycetoma: a review. Am J Clin Dermatol 7 5: 315–321. 17007542 10.2165/00128071-200607050-00005

[8] Magana M (1984) Mycetoma. Int J Dermatol 23 4: 221–236. 6376380 10.1111/j.1365-4362.1984.tb01238.x

[9] Brownell I, Pomeranz M, Ma L (2005) Eumycetoma. Dermatol Online J 11 4: 10. 16403382

[10] Negroni R, Lopez Daneri G, Arechavala A, Bianchi MH, Robles AM (2006) Clinical and microbiological study of mycetomas at the Muñiz Hospital of Buenos Aires between 1989 and 2004. Rev Argent Microbiol 38 1: 13–18. 16784127

[11] WHO (2013) The 17 neglected tropical diseases. Geneva: World Health Organization. Available: http://www.who.int/neglected_diseases/diseases/en/. Accessed 10 July 2014.

[12] de Hoog GS, van Diepeningen AD, Mahgoub e-S, van de Sande WW (2012) New species of *Madurella*, causative agents of black-grain mycetoma. J Clin Microbiol 50: 988–994. 22205798 10.1128/JCM.05477-11 PMC3295137

[13] van de Sande WWJ (2013) Global burden of human mycetoma: a systematic review and meta-analysis. PLoS Negl Trop Dis 7: e2550. 24244780 10.1371/journal.pntd.0002550 PMC3820768

[14] van de Sande WWJ, Maghoub ES, Fahal AH, Goodfellow M, Welsh O, et al (2014) The mycetoma knowledge gap: identification of research priorities. PLoS Negl Trop Dis 8: e2667. 24675533 10.1371/journal.pntd.0002667 PMC3967943

[15] Ezaldeen EA, Fahal AH, Osman A (2013) Mycetoma herbal treatment: the Mycetoma Research Centre, Sudan experience. PLoS Negl Trop Dis 7: e2400. 23991244 10.1371/journal.pntd.0002400 PMC3749975

[16] Fahal AH (2013) The Mycetoma Research Center, University of Khortum, Sudan: a success story that need support. Int J Sudan Res 3: 1–13.

[17] Abd El-Bagi ME, Fahal AH (2009) Mycetoma revisited: incidence of various radiographic signs. Saudi Med J 30: 529–533. 19370281

[18] de Hoog GS, Ahmed SA, Najafzadeh MJ, Sutton DA, Keisari MS, et al (2013) Phylogenetic findings suggest possible new habitat and routes of infection of human eumyctoma. PLoS Negl Trop Dis 7: e2229. 23696914 10.1371/journal.pntd.0002229 PMC3656121

[19] Fahal AH, Hassan MA (1992) Mycetoma. Br J Surg 79 11: 1138–1141. 1467883 10.1002/bjs.1800791107

[20] Fahal AH (2004) Mycetoma: a thorn in the flesh. Trans R Soc Trop Med Hyg 98: 3–11. 14702833 10.1016/s0035-9203(03)00009-9

[21] van Belkum A, Fahal A, van de Sande WW (2013) Mycetoma caused by *Madurella mycetomatis*: a completely neglected medico-social dilemma. Adv Exp Med Biol 764: 179–189. 23654067 10.1007/978-1-4614-4726-9_15

[22] Ahmed AOA, van Leeuwen W, Fahal A, van de Sande W, Verbrugh H, et al (2004) Mycetoma caused by *Madurella mycetomatis*: a neglected infectious burden. Lancet Infect Dis 4 9: 566–574. 15336224 10.1016/S1473-3099(04)01131-4

[23] Wieczorek J, Guo Q, Hijmans R (2004) The point-radius method for georeferencing locality descriptions and calculating associated uncertainty. Int J Geogr Inf Syst 18: 745–767.

[24] Fahal A (2011) Mycetoma. Khartoum Med J 41: 514–523.

[25] ISRIC-World Soil Information IWS (2013) Soil property maps of Africa at 1 km. Available for download at www.isric.org.

[26] Samy AM, Campbell LP, Peterson AT (2014) Leishmaniasis transmission: distribution and coarse-resolution ecology of two vectors and two parasites in Egypt. Rev Soc Bras Med Trop 47: 57–62. 24603738 10.1590/0037-8682-0189-2013

[27] Coll C, Wan Z, Galve JM (2009) Temperature-based and radiance-based validations of the V5 MODIS land surface temperature product. J Geophys Res-Oc ATM 114: D20102.

[28] Lyapustin AI, Wang Y, Laszlo I, Hilker T, G.Hall F, et al (2012) Multi-angle implementation of atmospheric correction for MODIS (MAIAC): 3. Atmospheric correction. Remote Sens Environ 127: 385–393.

[29] Peterson AT, Soberón J, Pearson RG, Anderson RP, Martínez-Meyer E, et al.. (2011) Ecological Niches and Geographic Distributions. Princeton: Princeton University Press.

[30] Phillips SJ, Anderson RP, Schapire RE (2006) Maximum entropy modeling of species geographic distributions. Ecol Model 190: 231–259.

[31] Owens HL, Campbell LP, Dornak LL, Saupe EE, Barve N, et al (2013) Constraints on interpretation of ecological niche models by limited environmental ranges on calibration areas. Ecol Model 263: 10–18.

[32] Peterson AT, Papeş M, Soberón J (2008) Rethinking receiver operating characteristic analysis applications in ecological niche modeling. Ecol Model 213: 63–72.

[33] Warren DL, Glor RE, Turelli M (2008) Environmental niche equivalency versus conservatism: quantitative approaches to niche evolution. Evolution 62: 2868–2883. 18752605 10.1111/j.1558-5646.2008.00482.x

[34] Barve N, Barve V, Jiménez-Valverde A, Lira-Noriega A, Maher SP, et al (2011) The crucial role of the accessible area in ecological niche modeling and species distribution modeling. Ecol Model 222: 1810–1819.

[35] Aref IM, Atta H, Shahrani T, Mohamed A (2011) Effects of seed pretreatment and seed source on germination of five *Acacia* spp. Afr J Biotechnol 10: 15901–15910.

[36] Kaempfer E (1694) Disputatio physica medica inauguralis exhibens decadem observationem exoticarum [phD thesis]. Netherlands: Univeristy of Leiden.

[37] Gokhale BB (1981) Epidemiology of mycetoma. Hindustan Antibiot Bull 23: 18–24. 7309550

[38] Peterson AT, Pereira RS, Neves VF (2004) Using epidemiological survey data to infer geographic distributions of leishmaniasis vector species. Rev Soc Bras Med Trop 37: 10–14. 15042174 10.1590/s0037-86822004000100003

[39] Escobar LE, Peterson AT, Favi M, Yung V, Pons DJ, et al (2013) Ecology and geography of transmission of two bat-borne rabies lineages in Chile. PLoS Negl Trop Dis 7: e2577. 24349592 10.1371/journal.pntd.0002577 PMC3861194

[40] Mak S, Klinkenberg B, Bartlett K, Fyfe M (2010) Ecological niche modeling of *Cryptococcus gattii* in British Columbia, Canada. Environ Health Persp 118: 653–658. 10.1289/ehp.0901448 PMC2866681 20439176

[41] Reed KD, Meece JK, Archer JR, Peterson AT (2008) Ecologic niche modeling of *Blastomyces dermatitidis* in Wisconsin. PLoS ONE 3: e2034. 18446224 10.1371/journal.pone.0002034 PMC2323575

[42] Ahmed AO, Desplaces N, Leonard P, Goldstein F, De Hoog S, et al (2003) Molecular detection and identification of agents of eumycetoma: detailed report of two cases. J Clin Microbiol 41: 5813–5816. 14662990 10.1128/JCM.41.12.5813-5816.2003 PMC309011

[43] Abd El-Bagi ME, Abdul Wahab O, Al-Thagafi MA, El-Sheikh H, Al-Salman K, Taifoor MK, Osman FM (2004) Mycetoma of the hand. Saudi Med J 25: 352–354. 15048175

[44] Ahmed AO, van Vianen W, ten Kate MT, van de Sande WW, van Belkum A, et al (2003) A murine model of *Madurella mycetomatis* eumycetoma. FEMS Immunol Med Microbiol 37 1: 29–36. 12770757 10.1016/S0928-8244(03)00096-8

[45] Maiti PK, Bandyopadhyay D, Dey JB, Majumdar M (2003) Mycetoma caused by a new red grain mycetoma agent in two members of a family. J Postgrad Med 49: 322–324. 14699230

[46] Chufal SSTN, Gupta MK (2012) An approach to histology-based diagnosis and treatment of Madura foot. J Infect Dev Ctries 6 9: 684–688. 23000870 10.3855/jidc.2387

[47] Maiti PK, Ray A, Bandyopadhyay S (2002) Epidemiological aspects of mycetoma from a retrospective study of 264 cases in West Bengal. Trop Med Int Health 7 9: 788–792. 12225511 10.1046/j.1365-3156.2002.00915.x

