# Supplementary material for: Mapping the Potential Risk of Mycetoma Infection in Sudan and South Sudan Using Ecological Niche Modeling
Source: PLoS Negl Trop Dis. 2014 Oct 16;8(10):e3250. doi: 10.1371/journal.pntd.0003250 (PMC4199553; doi:10.1371/journal.pntd.0003250)
Supplement: Text S1 — The variables of the soil characteristics used in model calibration for mycetoma and Acacia spp. in Sudan. Data downloaded from the World Soil Information (http://www.isric.org). Each variable is available in 2 depths (0–5 cm and 5–15 cm). (DOC) [file pntd.0003250.s001.doc]

**Text S1. The variables of the soil characteristics used in model calibration for mycetoma and *Acacia* spp. in Sudan.** Data downloaded from the World Soil Information ([http://www.isric.org](http://www.isric.org/)). Each variable is available in 2 depths (0-5 cm and 5-15 cm).

| **Soil organic carbon in permilles (g/kg) = ORCDRC** |
| --- |
| predicted mean value for the first standard depth (0-5 cm) |
| predicted mean value for the first standard depth (5-15 cm) |
| **pH in H2O 1:5 = PHIHO5** |
| predicted mean value for the first standard depth (0-5 cm) |
| predicted mean value for the first standard depth (5-15 cm) |
| **Sand content (50-2000 μm) in % = SNDPPT** |
| predicted mean value for the first standard depth (0-5 cm) |
| predicted mean value for the first standard depth (5-15 cm) |
| **Silt content (2-50 μm) in % = SLTPPT** |
| predicted mean value for the first standard depth (0-5 cm) |
| predicted mean value for the first standard depth (5-15 cm) |
| **Clay content (<2 μm) in % = CLYPPT** |
| predicted mean value for the first standard depth (0-5 cm) |
| predicted mean value for the first standard depth (5-15 cm) |
