# Supplementary material for: Mapping the Potential Risk of Mycetoma Infection in Sudan and South Sudan Using Ecological Niche Modeling
Source: PLoS Negl Trop Dis. 2014 Oct 16;8(10):e3250. doi: 10.1371/journal.pntd.0003250 (PMC4199553; doi:10.1371/journal.pntd.0003250)

**Text S2. Potential mycetoma distribution based on occurrences across all of Sudan.** These models were calibrated across all of Sudan directly based on all records collected from scientific literature and environmental variables for all of Sudan.


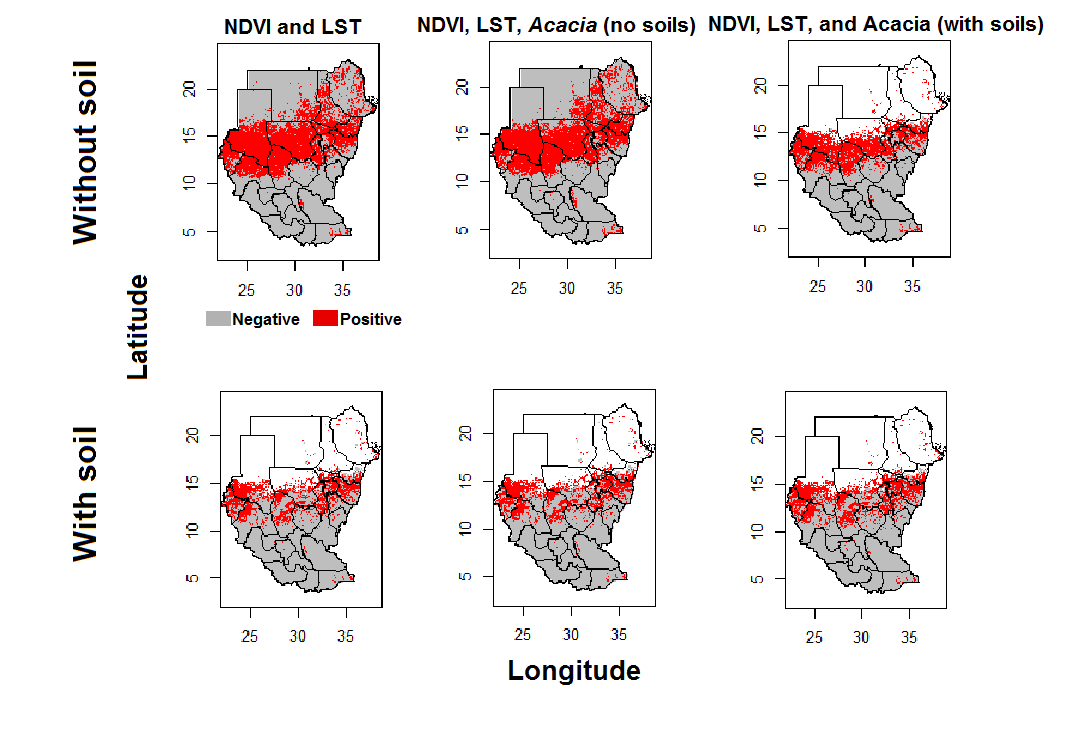

Supplement: Text S2 — Potential mycetoma distribution based on occurrences across all of Sudan. These models were calibrated across all of Sudan directly based on all records collected from scientific literature and environmental variables for all of Sudan. (DOC) [file pntd.0003250.s002.doc]
